# Signal Smoothing and Syntactic Choices: A Critical Reflection on the UID Hypothesis

**DOI:** 10.1162/opmi_a_00125

**Published:** 2024-03-05

**Authors:** Tom S. Juzek

**Affiliations:** Department of Modern Languages and Linguistics, Florida State University, Tallahassee, FL, USA

**Keywords:** cognitive linguistics, syntax, Uniform Information Density hypothesis, Smooth Signal Redundancy Hypothesis, syntactic alternations, surprisal, communicative efficiency

## Abstract

The Smooth Signal Redundancy Hypothesis explains variations in syllable length as a means to more uniformly distribute information throughout the speech signal. The Uniform Information Density hypothesis seeks to generalize this to choices on all linguistic levels, particularly syntactic choices. While there is some evidence for the Uniform Information Density hypothesis, it faces several challenges, four of which are discussed in this paper. First, it is not clear what exactly counts as uniform. Second, there are syntactic alternations that occur systematically but that can cause notable fluctuations in the information signature. Third, there is an increasing body of negative results. Fourth, there is a lack of large-scale evidence. As to the fourth point, this paper provides a broader array of data—936 sentence pairs for nine syntactic constructions—and analyzes them in a test setup that treats the hypothesis as a classifier. For our data, the Uniform Information Density hypothesis showed little predictive capacity. We explore ways to reconcile our data with theory.

## INTRODUCTION

Human language conveys, on average, a constant amount of information^1^ per time unit (Fenk & Fenk, 1980; Genzel & Charniak, 2002), and that amount is about the same across languages (Coupé et al., 2019). Various linguistic levels are used to modulate the flow of information. Most notably, in spoken language, the duration of syllables is adjusted such that peaks and troughs of information density are smoothed out. The duration of highly predictable words is reduced, and vice versa. This effect is described by the Smooth Signal Redundancy Hypothesis (*SSHR*) in Aylett (1999) and Aylett and Turk (2004). Recent evidence for this hypothesis can be found in the works of Priva (2017) and Tang and Shaw (2021). There is also evidence that information density is modulated in a similar fashion through morphological choices (Hanique & Ernestus, 2012; Kuperman et al., 2007) and lexical alternatives (Jurafsky et al., 2001; Mahowald et al., 2013; Piantadosi et al., 2011). Further, filler words such as *uhm* and *erm* are typically inserted in contexts where information density is high (Sen, 2020). The Uniform Information Density (UID) hypothesis extends these ideas to syntactic choices (Jaeger, 2006, 2010; Levy & Jaeger, 2006). The SSRH and the UID hypothesis state the following:

SSRH: “There is an inverse relationship between language redundancy and acoustic redundancy (as manifested by syllable duration)” (Aylett & Turk, 2004, p. 34).

UID: “Within the bounds defined by grammar, speakers prefer utterances that distribute information uniformly across the signal (information density). Where speakers have a choice between several variants to encode their message, they prefer the variant with more uniform information density (ceteris paribus)” (Jaeger, 2010, p. 25).

There are two important points to be made. First, the present paper is concerned with language production. What are the factors that guide a person in their syntactic choices when producing language? The hypothesis can also be adjusted for language perception. For example, Sikos et al. (2017) showed that a spoken non-uniform signal correlates with higher reading times. This was confirmed by Meister et al. (2021). Collins (2014) showed a relationship between information structure and linguistic acceptability, Meister et al. (2021) confirmed this for both grammaticality and acceptability.

Second, while the hypothesis is qualified by the use of *ceteris paribus*, in practice, there are very few syntactic alternations that do not affect other factors. That is, in assessing the hypothesis, information density is a factor that is almost always in competition with other factors. For instance, even one of the most prominent examples used in support of the UID hypothesis, optional *that* (Jaeger, 2006, 2010), affects prosody and rhyme^2^. Accordingly, the original studies in favor of the UID hypothesis provided multi-factorial analyses (Baayen et al., 2008). Syntactic choices, such as optional *that* or optional *to*, also slightly affect processing/memory load. However, there are other alternations that have a greater effect on processing load, for example, particle verbs with a longer verb-particle dependencies, as in *turn the music down* vs. *turn down the music*. The example is from Stone et al. (2020); for related research, see also Gibson (1998, 2001), Lohse et al. (2004), and Futrell et al. (2020). Other common alternations also have semantic consequences, for example topicalization (Nakanishi, 2005). They can have also sociolinguistic consequences; for example, non-canonical word order can be used to establish an informal context (Farrar, 1999).

In the more than 15 years since its inception, there has been a growing body of evidence to support the UID hypothesis. First, there are the original papers in support of it. These papers examined certain syntactic alternations, namely optional *that*-complements (Jaeger, 2006, 2010), optional *that*-conjunctions (Jaeger, 2006), and—on a morpho-syntactic level—also the use of contractions (Frank & Jaeger, 2008). In these studies, information density was identified as one of the most important factors in explaining syntactic alternations. Considering its general formulation, this is often interpreted as the UID hypothesis being a pervasive factor in a speaker’s syntactic choices.

Other studies, for example Demberg et al. (2012) corroborated the UID hypothesis, and studies such as Wasow et al. (2015) also provided some support, identifying information as one of the relevant factors for certain linguistic choices. Prosody had a greater influence, however. The number of challenges to the hypothesis has also been increasing. In the following section, we discuss four of them in greater detail. In our view, the main issue in this debate is the lack of a broader array of data, which is covered by the fourth challenge. Consequently, we collected more data and used a test setup in which we treated the hypothesis as a classifier (see More Quantitative Data section). The predictions made by the UID hypothesis were sub-optimal, and the paper closes by exploring ways to reconcile data and theory.

## FOUR CHALLENGES

### No Exact Definition

In Jaeger (2006, 2010), smoothing was defined as avoiding peaks and troughs throughout the speech signal. This is measured through the sum of absolute differences in surprisal of word pairs, as per Equation 1, where there are *n* words in a sequence, and *s*(*w*) is a word’s surprisal value. In the following, we will refer to this as Δ-*UID*.

$$\left(\Delta ‐UID\right)\;\sum_{i=1}^{n}\left|s\left(w_{i}\right)-s\left(w_{i-1}\right)\right|$$ (1)

**Figure 1.**
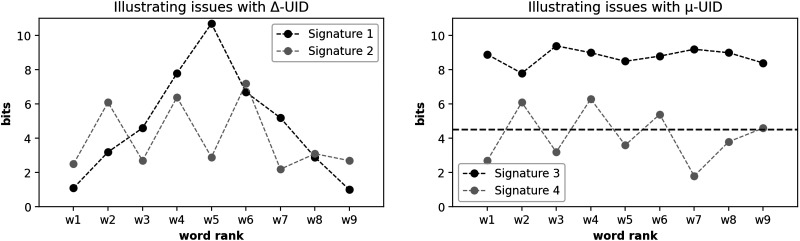
Hypothetical information signatures to illustrate issues with different interpretations of a “uniform” information structure. The dotted line on the right represents an approximation of a global average of word surprisal for human communication in general. The contrast between Signatures 1 and 2 is inspired by Figure 4 in Collins (2014) and Figure 2 in Meister et al. (2021).

As Collins (2014) and later Meister et al. (2021) observed, Δ-*UID* makes certain predictions. These predictions are not necessarily problematic, but it is important to be aware of them. A gradually changing, but extreme information signal would be preferred to a signature that fluctuates around a certain mean. Figure 1 (left) illustrates this: Δ-*UID* prefers Signature 1 to Signature 2, despite Signature 1’s extreme endpoints^3^. Thus, Meister et al. (2021) suggested what we call *μ*-*UID*, in which the mean information content of a sequence is measured, and then for the sequence’s words, the differences from the mean are summed up. This is expressed in Equation 2, where *s*(*seq*) is the average information value of the sequence in question.

$$\left(\mu ‐UID\right)\;\sum_{i=1}^{n}\left|s\left(seq\right)-s\left(w_{i}\right)\right|$$ (2)

Comparing a sequence with smooth but extremely high information density to a sequence with fluctuating but average information density, *μ*-*UID* favors the former. This preference is illustrated in Figure 1 (right), where *μ*-*UID* selects Signature 3 over Signature 4, despite the high surprisal values observed throughout the signal of Signature 3. Although this preference is not inherently problematic, either, it is important to acknowledge the potential implications and precise predictions of the various *μ*-*UID* variants in this scenario.

An alternative take could be to measure a word’s difference in surprisal to a global mean, that is, an approximation of the mean surprisal of all human communication. On average, a human word transmits between 4 to 5 bits of information, depending on the dataset. Taking the average of a corpus is in our view a reasonable approximation. This is expressed in Equation 3, where $\bar{s}$ is the average surprisal value for a corpus or a set of corpora.

$$\left(RTM\right)\;\sum_{i=1}^{n}\left|\bar{s}-s\left(w_{i}\right)\right|$$ (3)

The corresponding hypothesis is whenever speakers face a choice, they choose the alternative with the lowest sum of differences to the global mean. This is what we call the regression to the mean *RTM* hypothesis, and it is similar to the idea of a constant entropy rate in Genzel and Charniak (2002). Importantly, it is far from trivial to give a solid definition for what exactly it means for syntactic choices to smooth a sequence’s information structure. This contrasts with the simplicity of the SSRH, the predictions of which are very clear.

In More Quantitative Data section, we put these hypotheses to a test. For this purpose, we introduce a baseline, which is simply reducing a sequence’s average surprisal value, which we refer to as the low information content *Low-IC* model.

### High Surprisal Alternatives

In More Quantitative Data section, we will present quantitative data on nine syntactic alternations. In the current subsection, we will briefly examine the usage of verb-second *weil* (“because”) in German, to gain some broader insights. The analysis is informal and is included for illustration purposes (instead of relying on entirely theoretical or constructed examples). The analysis was done with a lexical trigram model trained on the Dortmund Chat Korpus (Lüngen et al., 2017).

Typically, *weil* requires a verb-final word order, as demonstrated in Sequence (1). However, it has been observed that under certain circumstances, speakers use verb-second structures (Kempen & Harbusch, 2016). Sequence (2) provides an example of such a non-canonical word order.

(1)  Die Situation ist nicht so gut, weil ich nicht zahlen kann.
    The situation is not so good because I not pay can.
    “The situation is not so good because I cannot pay.” (canonical; 46.7 bits)
(2)  Die Situation ist nicht so gut, weil ich kann nicht zahlen.
    The situation is not so good because I can not pay.
    “The situation is not so good because I cannot pay.” (non-canonical; 52.2 bits)

The factors determining the usage of verb-final versus verb-second are complex (Antomo & Steinbach, 2010). Verb-second usage can serve as a discourse marker (Gohl & Günthner, 1999) or indicate an informal setting (Farrar, 1999). Reducing overly long dependencies (Hawkins, 1994, 2004) might also play a role. We extracted 469 sequences with a *weil* from the Dortmund Chat Korpus (Lüngen et al., 2017), of which 24 (5%) had a verb-second *weil*. 17 of the 24 verb-second sentences (71%) would have had a smoother information signature if the *weil* had been in the canonical word-final form.

**Figure 2.**
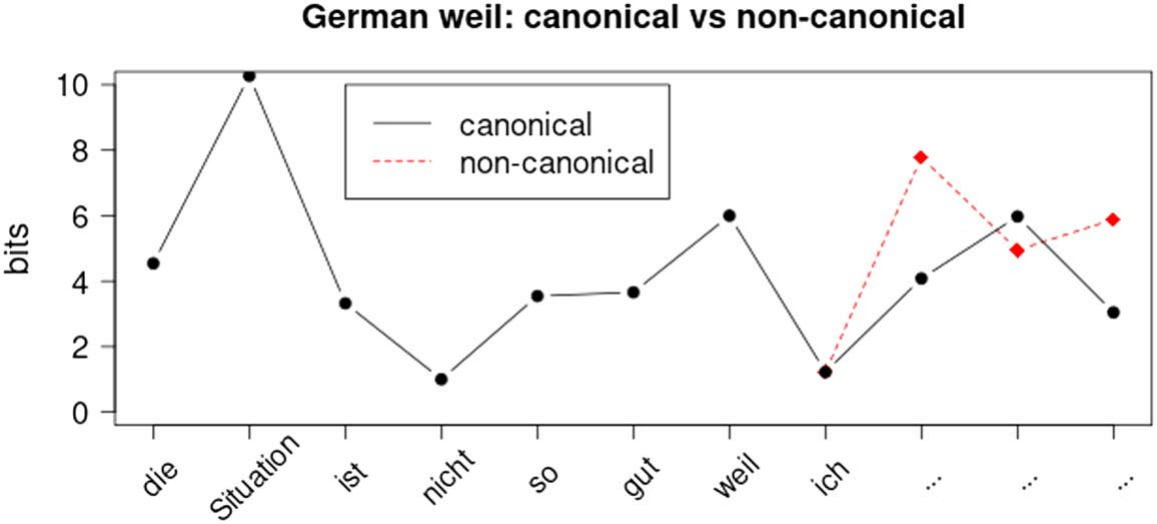
Non-canonical alternatives generally lead to less well-distributed information signatures, but they occur regularly.

Importantly, models based solely on word-surprisal measures predict that non-canonical word orders generally result in spikes in information density, causing the signal to become less uniform, as illustrated in Figure 2. However, the issue might be due to practical considerations, i.e. data issues, rather than theoretical considerations, i.e. issues surrounding the UID hypothesis itself. If our data were to incorporate socio-pragmatic or other suprasegmental factors, our models could potentially make more accurate predictions than those based solely on word-surprisal. In the example of *weil*, if we had setting (*weil*-informal versus *weil*-formal) or social distance (*weil*-speaker-familiar versus *weil*-speaker-unfamiliar) somehow encoded, then the predictive accuracy might potentially improve. This limitation does not reflect an inherent problem with the UID hypothesis; on the contrary, our interpretation of Jaeger’s original work suggests that an extension to other linguistic levels is desired. If anything, practical issues (e.g., difficulties in collecting and quantifying such information) hinder this extension. In our view, more evidence is needed regarding such considerations. We decided to include this issue, because ultimately, these are considerations one has to take into account when working with information structure.

### Negative Evidence

In recent years, several studies have reported negative results for the UID hypothesis. That is, the factors under investigation could not be explained with considerations around information distribution. Horch and Reich (2016) analyzed optional article placement in German, with mixed results. At the word level, article omission even leads to less well-distributed structures; at the part-of-speech level, the distribution is more uniform. Ranjan et al. (2020) looked at various alternations in English involving word order, namely the dative alternation, what they call the quotation alternation, and pre- vs. post-verbal adjuncts. In their analyses, they used both lexical and syntactic surprisal. However, their results were contrary to what the UID hypothesis would predict. Jain et al. (2018) tested word order alternation in Hindi, also using both lexical and syntactic surprisal, and they, too, did not find evidence for the predictions the UID hypothesis makes. Zhan and Levy (2018) looked at the effects of information structure on optional classifiers in Mandarin Chinese, and their results were also not in support of the UID hypothesis.

There are considerably more studies with supporting evidence available (Demberg et al., 2012; Jaeger, 2006, 2010, 2011; Kurumada & Jaeger, 2013, 2015; Levy & Jaeger, 2006; Temperley & Gildea, 2015), inter alia, than there are studies with mixed (Horch & Reich, 2016; Juzek & Bizzoni, 2021; Yu et al., 2016) or even negative (Jain et al., 2018; Ranjan et al., 2020; Zhan & Levy, 2018) results, with a more than three-to-one ratio for positive vs. null results reported. On GitHub (https://www.github.com/tjuzek/om-uid), we maintain a table that provides an overview of the literature.

However, it is important to keep in mind that negative results are affected by a publication bias and that the ratio of not-published vs. published results is higher for negative results than for positive ones (Dickersin, 1990; Ferguson & Heene, 2012; Hubbard & Armstrong, 1997). By some estimates, more than 90% of the results published in psychology/psychiatry are positive (Fanelli, 2010). Franco et al. (2014) estimated that in the social sciences, only one in five negative results get published, while three out of five positive results get published. That means, for every negative result that is published, there are on average another four in the drawer; for every positive result published, this number stands at about 0.6.

Further, it is significant that mixed and negative evidence comes from various groups, concerns multiple languages, and a variety of syntactic alternations. Of course, there is the question of what to do with negative outcomes in general. The absence of evidence is not evidence of absence (Altman & Bland, 1995). None of the studies with negative results *disprove* the UID hypothesis. Still, there is value in null results (Kepes et al., 2014), and an explanation that reconciles these results is needed.

### Lack of Large-Scale Evidence

All studies in support of the UID hypothesis guiding a speaker’s production choices have examined isolated phenomena, most notably some instances of optional *to* and optional *that*. However, there has not been any large-scale, multi-language evidence so far. As for language perception, Meister et al. (2021) have provided large-scale evidence that higher reading times are correlated with high surprisal, in a super-linear manner, and that ungrammatical sequences discussed in the linguistic literature have higher surprisal (these ungrammatical sequences are not choices speakers typically consider in language production, though). Research from related areas could also provide guidance as to how such large-scale evidence could look. For instance, Liu (2010) examined typological features across 20 languages, and Futrell et al. (2015) analyzed 37 languages to test the Dependency Locality Hypothesis (Gibson, 1998, 2001). Importantly, this has been attempted for the UID hypothesis. The approach in Jain et al. (2018) went beyond single constructions, but their results were negative. Similarly, Juzek and Bizzoni (2021) tested syntax-semantics interactions at a larger scale, examining 38 languages. If their results had been positive, this could have been viewed in favor of the UID hypothesis, but their results were also negative. The same holds for results in Yu et al. (2016), who looked at information structure in the British National Corpus, and their results were also not in support of the UID hypothesis.

## MORE QUANTITATIVE DATA

The lack of large-scale evidence is in our view the biggest issue in the debate regarding the UID hypothesis. What is needed is a broader dataset, with more syntactic constructions across multiple languages. For this paper, we took a step into that direction; however, we began with English-language data only.

### Materials

We compiled a list of syntactic alternations in which speakers could make a syntactic choice between two variants^4^. Nine syntactic constructions had a reasonable cost–return ratio regarding data collection. See Table 1 for a list. Items were extracted from various corpora with Python scripts and then manually checked. Where present, special characters and final punctuation were removed. Items were extracted with as much context as possible. We used utterance boundaries as cut-off points. Items obtained through existing research tended to consist of single sentences. Each construction’s average GPT token length is also provided in Table 1. This roughly corresponds to the average sentence length minus one. For example, the average sentence length of items with cataphora and their counterparts is about 24 words.

**Table 1.** Information on the nine syntactic alternations for which we were able to collect corpus data.

	**Do-be *to***	***That*-relativizer drop**	**Cataphora**	**Extraposition**	**Topicalization**	**Double-NP dative**	***Seem*-raising**	**Sluicing**	***Tough*-raising**
**Surface change**	Syllabic	Syllabic	Word order	Word order	Word order	Complex	Complex	Complex	Complex
**Source**	COCA	BROWN, LOB, FLOB, FROWN	LCC	ICE	Switchboard	Switchboard	Switchboard	LCC	LCC
**Tokens**	App. 450 m	App. 4 m	App. 1085 m	App. 1 m	App. 0.3 m	App. 0.3 m	App. 0.3 m	App. 1085 m	App. 1085 m
**Mode**	Mixed	Written	Mixed	Mixed	Spoken	Spoken	Spoken	Mixed	Mixed
**Via**	Flickinger and Wasow (2013), Wasow et al. (2015), Melnick and Wasow (2019)	Hinrichs et al. (2015), Grafmiller et al. (2018)	Extracted	Francis (2010), Francis and Michaelis (2014, 2017)	Extracted	Extracted	Extracted	Extracted	Extracted
***N* (pairs)**	255	82	100	69	118	83	69	93	67
***N* critical (pairs)**	124	41	50	37	58	55	38	49	45
***N* baseline (pairs)**	131	41	50	32	60	28	31	44	22
**Avg. GPT tokens**	17.0	34.6	23.2	26.7	14.5	15.9	28.7	20.3	22.5

For each construction, we collected corpus occurrences of the critical condition as well as baseline examples. For example, Sequence 3 from the Leipzig Corpora Collection (LCC) for English (Goldhahn et al., 2012) is a real instance of sluicing, and Sequence 4, also from the LCC, is a real instance of a non-sluiced sequence. Data for the *do-be* construction came from Flickinger and Wasow (2013), Wasow et al. (2015), and Melnick and Wasow (2019). Data for *that*-relativizers came from Hinrichs et al. (2015) and Grafmiller et al. (2018), and data for the extrapositions came from Francis (2010) and Francis and Michaelis (2014, 2017).

(3)  We wrestled way better than I thought we would. (actual sluicing)
(4)  Our economies can’t really breathe the way they should breathe. (actual full-form)

We then constructed hypothetical counterparts, giving us syntactic minimal pairs. For critical items, where a syntactic phenomenon is realized, we constructed hypothetical baselines, and for actual baseline items, we constructed hypothetical critical items. The hypothetical baseline for Sequence 3 is Sequence 5, and the hypothetical critical counterpart of Sequence 4 is Sequence 6. For about half of the pairs, the critical item was the item that actually occurred, and for the other half, the baseline was actually produced. The constructed counterparts were checked by a native speaker^5^.

(5)  We wrestled way better than I thought we would wrestle. (constructed full-form)
(6)  Our economies can’t really breathe the way they should. (constructed sluicing)

Examples for the other constructions can be found in Sequences 7 to 14. Our dataset is accessible on GitHub (https://www.github.com/tjuzek/om-uid).

(7)  All you have to do is just (to) do a basic stretch. (do-be optional to)
(8)  It was a mental game (that) he had started playing recently. (that-relativizer drop)
(9)  When she begins a new work, Freeman … (*vs*. When Freeman begins …) (cataphora)
(10)  However new sets soon appeared that were able to receive all the TV channels (extraposition) (*vs*. However new sets that were able to receive all the TV channels soon appeared)
(11)  Now to me that is inhumane. (*vs*. Now that is inhumane to me.) (topicalization)
(12)  Maybe we can send you some. (*vs*. … send some to you) (double-NP dative)
(13)  It seems that the electorate is … (*vs*. The electorate seems to be …) (seem-raising)
(14)  It is tough to stop deaths … (*vs*. To stop deaths … is tough) (tough-raising)

We should note that there is a selection bias in our materials. Difficult-to-construct counterparts in either direction were skipped, such as cases where constructing a baseline counterpart for extrapositions was challenging or when an extraposition was not a viable option for a sentence in canonical word order. This practical approach ensures a focus on meaningful alternations rather than including imbalanced non-choices. Arguably, including more of imbalanced “alternatives” could have had a favorable effect on the predictive accuracy of Δ-*UID* and *μ*-*UID*, but we would expect an even greater impact on the Low-IC model.

Furthermore, our data come from corpora of different modalities: written versus spoken, or even mixed modality. We expect that the predictions of the different frameworks are most accurate for spoken language, as written language is less susceptible to cognitive pressures and allows for editing. As a consequence, we will briefly discuss results for spoken data separately.

### Obtaining Surprisal

We needed to obtain surprisal values for our items. In the examples in No Exact Definition section, we used trigram models for surprisal. For illustrative purposes, such simple models are sufficient, but compared to more powerful models, results can differ, as illustrated in Wei et al. (2021). Thus, the results in this section are based on surprisal calculated with OpenAI’s Generative Pre-trained Transformer 3.5 (GPT-3.5), as per Brown et al. (2020).

We decided to obtain surprisal with GPT since surprisal and perplexity values from Transformer models align with the results from N-gram models and often exhibit increased performance across various tasks. Several studies have indirectly verified this; see, for example, Figure 1 in Wilcox et al. (2020), where GPT-2 produces similar results to a 5-gram model. Similarly, refer to Figure 2 in Kuribayashi et al. (2021), or to Hao et al. (2020), who observe that “GPT-2 outperforms all other models on all metrics” (including an N-gram model). Additionally, Hu et al. (2020) note that “the bestperforming model (GPT-2-XL) (scores) over twice as high as the worst-performing model” (also including an N-gram model). In general, GPT-3.5 performs well in various cognitive tasks of linguistic relevance (Cai et al., 2023). However, we are not aware of a direct *validation*, testing N-gram model surprisal versus Transformer-based surprisal. Furthermore, one possible issue arises from the fact that GPT-3.5 was mostly trained on written language. Ideally, for our materials, which come from both written, spoken, and mixed corpora, we would use a Large Language Model that was trained on a larger amount of spoken data.

We use the third iteration of the DaVinci model (“text-davinci-003”) and the Python implementation by Sathe (2022). Among the models suitable for surprisal extraction, text-davinci-003 stands out as the one with the largest training dataset. However, it is important to note that text-davinci-003 underwent fine-tuning on instructional input. We also conducted analyses using GPT-2 (Radford et al., 2019), and the findings from both GPT-2 and GPT-3.5 come out similar, with most frameworks making slightly better predictions based on GPT-3.5 surprisal. Therefore, in the following we primarily focus on the surprisal values obtained from GPT-3.5. For calculating surprisal with GPT-2, we utilized the implementation by Misra (2022). The detailed GPT-2 results, along with a corresponding plot, can be accessed on GitHub (https://www.github.com/tjuzek/om-uid).

### Test Setup

We then tested the sentence pairs (actual occurrences vs hypothetical counterparts) with respect to the different interpretations of the UID hypothesis, Δ-*UID* and *μ*-*UID*, as per Equations 1 and 2; the RTM alternative, as per Equation 3; and to Low-IC (normalized for sentence length where applicable). As such, we treat the hypotheses as simple, deterministic classifiers to make *post factum* predictions: When presented with the two variants, one of which has actually occurred, the other being a constructed counterpart, which one do you prefer? In this regard, decisions pertaining to each pair are determined by selecting the variant that performs ‘better’ according to the relevant metric. We did this for the critical conditions and their baselines.

For example, Sequence 15 (the actual occurrence) and Sequence 16 (its constructed counterpart) constitute a pair. We obtained the surprisal values for the sequences and then examined the predictions made by each framework. For instance, the fluctuations in surprisal were lower for Sequence 16 than for Sequence 15. In Sequence 15, the average fluctuation as per Δ-UID was 5.99 bits, while in Sequence 16, it is 3.01 bits. As a result, Δ*-UID* incorrectly predicted that Sequence 16 was preferable; in other words, it incorrectly predicted that Sequence 16 was the sequence that had been actually produced.

*μ*-UID is operationalized as follows:For each sentence, the average information content is calculated. For example, it is 4.63 bits for Sequence 15 and 8.10 bits for Sequence 16. Then, the differences from a sequence’s average are summed, and this sum of differences is normalized by the sentence length. For Sequence 15, the average divergence from a local mean is 2.82 bits. For Sequence 16, it is 1.50 bits. Hence, *μ*-UID also makes an incorrect prediction with respect to this sentence pair. The RTM model is calculated in a similar way to *μ*-UID. However, instead of computing differences relative to a sequence’s average information content, a global average is used (in this case, approximately 4.70 bits). The Low-IC model is fundamentally based on a sequence’s average information content, normalized for sentence length. It correctly predicts that Sequence 15 is preferable to Sequence 16, as (15)’s average information content is lower (on average 4.63 bits vs 8.10 bits).

(15)  It  is  tough to  score (*actual*)
    (5.33) (1.93) (7.47) (0.30) (8.14) (bits)
    Δ-UID: 5.99 = avg(abs(5.33 − 1.93) + abs(1.93 − 7.47) + abs(7.47 − 0.30) + abs(0.30 − 8.14))
    *μ*-UID: 2.82 = avg(abs(4.63 − 5.33) + abs(4.63 − 1.93) + abs(4.63 − 7.47) + abs(4.63 − 0.30) + …)
(16)  To  score is  tough (*constructed*)
    (6.76) (9.48) (6.45) (9.72) (bits)
    Δ-UID: 3.01 = avg(abs(6.76 − 9.48) + abs(9.48 − 6.45) + abs(6.45 − 9.72))
     *μ*-UID: 1.50 = avg(abs(8.10 − 6.76) + abs(8.10 − 9.48) + abs(8.10 − 6.45) + abs(8.10 − 9.72))

This test setup gives four possible results: a true positive (a critical condition has occurred, and the model predicts it correctly), a false positive (a baseline has occurred, but the model predicts critical condition), false negatives (etc.), and true negatives. With this, we could compute an effective metric: the F_1_-score (Chinchor, 1992; Van Rijsbergen, 1979), implemented as per Raschka and Mirjalili (2019, pp. 211–214). The F_1_-score is effective because for a balanced set in a binary classification task, it has a baseline that is easy to interpret. For a perfectly balanced dataset, a fair coin achieves a score of exactly 0.5. Our data are mostly well-balanced, a point we will address in Contextualization and Significance section. Good F_1_-scores are task and field dependent.

### Results

**Figure 3.**
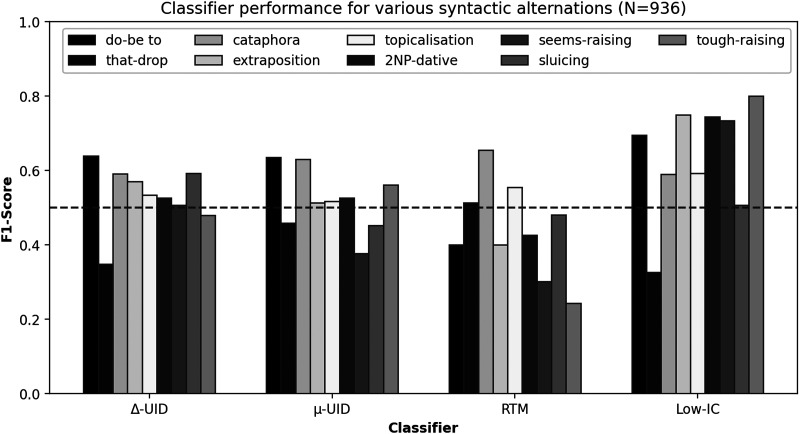
The results of letting the different models make predictions about syntactic alternations. The dashed line is the F_1_-score for a fair coin.

The results are illustrated in Figure 3, a colour-coded version can be found online in the GitHub repository. None of the models performed well. For five out of nine constructions, Δ-UID’s performance was below or around the random baseline (with an F_1_-score < 0.55), for only one constructions, the F_1_-scores were above 0.6. *μ*-UID’s overall performance was about as good or bad as random, and only two constructions have F_1_-scores above 0.6. The RTM model performed considerably worse than random, the Low-IC model had the best predictions, making decent predictions for five of the nine constructions. Notably, scores of 0.8 are possible^6^.

For the three phenomena that were extracted exclusively from spoken data—topicalizations, double-NP datives, and *seem*-raising—we observe near-random results for Δ-UID (scores of 0.53, 0.53, and 0.51, respectively) and better scores for the Low-IC model (0.59, 0.74, and 0.73, respectively).

### Contextualization and Significance

The results require contextualization to enhance interpretability. First, F_1_-Scores perform effectively with balanced data. Our sub-datasets for the various constructions are largely balanced. However, two imbalances exist: 55 critical pairs compared to 28 baseline pairs for the double-NP dative construction, and 45 pairs versus 22 pairs for the *tough*-raising construction. To aid readers in interpreting the scores, we introduce a data simulation for a Random Model.

**Figure 4.**
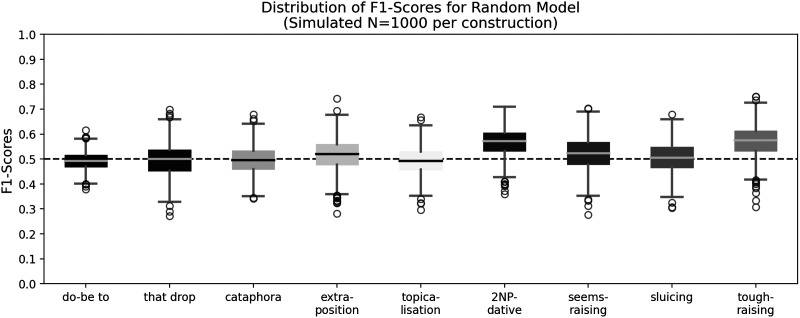
Box plots for simulating a Random Model, a 1000 times. The dashed line indicates the median of a perfectly balanced dataset.

For each of the 936 pairs, the Random Model randomly selects one of the two alternatives. We calculated the F_1_-Scores as described above, repeating the process 1000 times, resulting in 1000 F_1_-Scores for the Random Model. This provides basic statistical metrics, such as median and variance. The results of the data simulation are depicted in Figure 4. Generally, the median for most constructions is around 0.5, with the Third Quartile at approximately 0.55. The double-NP dative construction and the *tough*-raising construction are exceptions, where the median is roughly 0.57, and the Third Quartile is about 0.62. Repeating the simulation yields similar outcomes. This analysis underscores that F_1_-Scores struggle with imbalanced datasets (Chicco & Jurman, 2020; Davis & Goadrich, 2006).

Furthermore, we conducted significance tests. The most straightforward comparison involves assessing the F_1_-Scores of each model, as depicted in Figure 3, and contrasting them with the F_1_-Scores of a random baseline (*M* = 0.516, *SD* = 0.028). For this purpose, we employed one-sided paired *t* tests, focusing solely on whether a model has surpassed the performance of the random baseline. The outcomes are as follows: The Δ-UID model (*M* = 0.533, *SD* = 0.085) does not exhibit a significant improvement (*t*(8) = 0.545, *p* = 0.300); the *μ*-UID model (*M* = 0.519, *SD* = 0.084) also lacks a significant improvement (*t*(8) = 0.124, *p* = 0.452); similarly, the RTM model (*M* = 0.442, *SD* = 0.127) demonstrates no substantial enhancement, with *t*(8) = −1.520, *p* = 0.916. Notably, the Low-IC model (*M* = 0.638, *SD* = 0.150) is the only model that yields significant results (*t*(8) = 2.718, *p* = 0.013), underscoring that significant differences are achievable even with the limited sample size (comprising two sets of 9 values each).

To address the issue of a low *N*, we supplemented our analysis with a per-construction comparison, using a series of Chi-square tests. In this approach, we took the correct/incorrect responses from each model for every construction and compared them to random choices. The results are presented in Table 2.

**Table 2.** The results of the Chi-Square tests, per construction and per model, are compared to the random baseline. Outcomes in which a model performs significantly better than the random baseline are highlighted in **boldface**, while those with significantly worse performance are highlighted in italics. The degrees of freedom are consistently 1, and *N* values correspond to the “*N* (pairs)”-row in Table 1.

*Construction*	**Δ-UID**	***μ*-UID model**	**RTM**	**Low-IC**
**Do-be *to***	*χ*^2^ = 0.10, *p* = 0.754	*χ*^2^ = 1.13, *p* = 0.287	*χ*^2^ = 5.97, *p = 0.015*	*χ*^2^ = 10.2, ***p* = 0.001**
***That*-relativizer**	*χ*^2^ = 0.00, *p* = 1.000	*χ*^2^ = 3.12, *p* = 0.077	*χ*^2^ = 2.39, *p* = 0.122	*χ*^2^ = 0.78, *p* = 0.377
**Cataphora**	*χ*^2^ = 4.00, ***p*** = **0.046**	*χ*^2^ = 4.00, ***p*** = **0.046**	*χ*^2^ = 3.24, *p* = 0.072	*χ*^2^ = 13.0, ***p* = 0.000**
**Extraposition**	*χ*^2^ = 1.17, *p* = 0.279	*χ*^2^ = 0.01, *p* = 0.904	*χ*^2^ = 3.26, *p* = 0.071	*χ*^2^ = 19.8, ***p* = 0.000**
**Topicalization**	*χ*^2^ = 0.85, *p* = 0.357	*χ*^2^ = 7.63, ***p*** = **0.006**	*χ*^2^ = 0.31, *p* = 0.581	*χ*^2^ = 7.63, ***p* = 0.006**
**Double-NP dat.**	*χ*^2^ = 0.11, *p* = 0.742	*χ*^2^ = 0.59, *p* = 0.442	*χ*^2^ = 4.35, *p = 0.037*	*χ*^2^ = 14.8, ***p* = 0.000**
***Seem*-raising**	*χ*^2^ = 0.13, *p* = 0.718	*χ*^2^ = 0.13, *p* = 0.718	*χ*^2^ = 0.36, *p* = 0.547	*χ*^2^ = 10.6, ***p* = 0.001**
**Sluicing**	*χ*^2^ = 7.84, ***p*** = **0.005**	*χ*^2^ = 0.01, *p* = 0.917	*χ*^2^ = 2.42, *p* = 0.120	*χ*^2^ = 5.69, ***p* = 0.017**
***Tough*-raising**	*χ*^2^ = 1.81, *p* = 0.179	*χ*^2^ = 1.81, *p* = 0.179	*χ*^2^ = 16.3, *p = 0.000*	*χ*^2^ = 7.90, ***p* = 0.005**

As mentioned in Test Setup section, for datasets that are not perfectly balanced, F_1_-scores can have issues with interpretability. The subset for *tough*-raising illustrates this point effectively. Of the 67 cases of potential *tough*-raising that we were able to collect, the *tough* was raised in 45 cases, while in 22 cases it was not raised (see Table 1). On average, a fair coin would predict raising in 22.5 cases out of the 45 instances where *tough* was actually raised, and 11 cases of raising for the 22 instances where *tough* was not raised. Thus, on average, there are 22.5 true positives, 22.5 false negatives, 11 false positives, and 11 true negatives. This results in a precision of 0.67, a recall of 0.50, and it gives an F_1_-score of 0.57, which is exactly what we observe in Figure 4. Accordingly, we see slight issues with the F_1_-scores of the *tough*-raising and the double-NP dative constructions, other constructions are more well-balanced.

Thus, for additional interpretability, we offer further contextualization by comparing the accuracies of different models to the performance of a majority model. The majority model simply always chooses the alternative that is more frequent in our data. For example, in the case of *tough*-raising, it always chooses raising over non-raising. The majority model is a reasonable, easy to interpret baseline, and whenever another model outperforms the majority model, this indicates added value. The results are given in Table 3, and we observe that the Low-IC model is the only one that consistently outperforms the majority model.

**Table 3.** Accuracy results, per construction and per model, including a majority model, in italics. Results where another model outperforms the majority model are highlighted in **boldface**.

*Construction*	**Majority**	**Δ-UID**	***μ*-UID model**	**RTM**	**Low-IC**
**Do-be *to***	*0.51*	0.51	**0.53**	0.42	**0.60**
***That*-relativizer**	*0.50*	0.50	**0.60**	**0.59**	**0.55**
**Cataphora**	*0.50*	**0.60**	**0.60**	**0.59**	**0.68**
**Extraposition**	*0.53*	**0.57**	0.51	0.39	**0.77**
**Topicalization**	*0.51*	**0.54**	**0.53**	**0.53**	**0.63**
**Double-NP dat.**	*0.66*	0.48	0.46	0.39	**0.71**
***Seem*-raising**	*0.55*	0.52	0.52	0.46	**0.70**
**Sluicing**	*0.52*	**0.65**	0.51	0.42	**0.62**
***Tough*-raising**	*0.67*	0.42	0.42	0.25	0.67
**Average**	*0.55*	0.53	0.52	0.45	**0.64**

A multi-factorial analysis might provide additional valuable insights. However, we believe that prosody, as e.g., per Wasow et al. (2015), should be incorporated as one of the factors in such an analysis. While annotating prosodic information is beyond the scope of our current study, we think that including this information and subsequently reanalyzing the data would be a promising direction for future research.

### Discussion

For our data (*N* = 936 sentence pairs), we did not observe a positive effect of the desire to signal smoothing on syntactic choices in language production. We included Low-IC as a “baseline” model, and it made the best predictions. Critically, there is some systematicity and further analyses would have to explore relevant factors. However, Low-IC has little *predictive* value: It predicts that certain constructions are less likely to be produced because their surprisal values are high. Arguably, however, less frequent productions are the very reason why the surprisal values are high(er).

Ideally, even more syntactic constructions should be investigated, with a higher *N* (> 10 000) and with items from multiple languages. Furthermore, our materials are subject to the aforementioned selection bias, and there is something to be said about the combination of modalities. The UID hypothesis primarily focuses on spoken language, and we observe that the results obtained from the three phenomena extracted from spoken corpora were subpar. Ideally, however, we would have collected items for the nine constructions twice, once from spoken corpora and once from written corpora, and subsequently compared the results. Similarly, a more optimal test setup would involve a Large Language Model that incorporates a larger amount of training data from spoken language. As such, the presented results are just a step towards a clearer picture. However, under the assumption that the trends in our results are to some degree representative, we observe that the underlying linguistic reality is, as so often, complex.

There is still the question as to what to do with the existing positive evidence, particularly concerning certain instances of optional *to* and optional *that*, as per Jaeger (2006, 2010). The way we conceptualize such data is that we think of them as mono-syllabic phenomena, and the +/− realization could correspond to +/− syllable lengthening as predicted by the SSRH. This could be understood as a word-level manifestation of phonetic processes. This is a loose hypothesis, and more evidence is needed to solidify it. However, such an extension of the SSRH could bring together observations on various linguistic levels. Data from morphological contractions (*we’re* vs. *we are*), as reported by Frank and Jaeger (2008), could be viewed in this light, i.e., shortened vs. full syllable length. Furthermore, adding a filler word is understood as introducing a low information content syllable (Sen, 2020). Importantly, such phonetic manifestations on other linguistic levels would be a secondary effect, and other factors often take precedence, like memory limitations, social considerations, or pragmatic factors. The appeal of this hypothesis is that it explains the available data without making any claims for phenomena that came out negative, like cataphoras or extrapositions, in our analysis above.

Even if one does not agree with our reinterpretation, there is still value in our data. From a Kuhnian perspective (Kuhn, 1962), the UID hypothesis is the established paradigm and our data pose an anomaly. Either such anomalies can be reconciled with the existing framework(s) one way or the other, or they can be viewed as a bridge to somewhere else, to new, emerging frameworks. As such, we think that collecting and analysing more data in more detail is an important next step. Further, whatever one makes of our data, there is merit in the fact that the original work on the UID hypothesis has popularized information theoretic approaches for language analysis. This addition to a linguist’s toolkit has proven fruitful in various subareas of linguistics, in particular with respect to language perception.

## CONCLUDING REMARKS

The notion that information density would be a (if not the) major factor determining syntactic choices is a compelling one. However, there are four challenges to this idea, as discussed above. We presented data for nine syntactic constructions, all of which give speakers a choice, but signal smoothing is not a factor in many of the analysed choices. That is, information flow is modulated on other linguistic levels than syntax. At a minimum, our data pose an anomaly to the existing framework. As argued, the positive evidence might be subsumed under an extension of the Smooth Signal Reduction Hypothesis. As such, those processes could be viewed as phonetic cross-over effects. This is stipulated as a secondary effect, and more evidence is needed to confirm this.

Many factors have been shown to influence linguistic choices (our focus was on language production). As for syntactic choices, they are arguably governed by a mix of factors, including prosody and stress (Gries, 2007; Melnick & Wasow, 2019; Wasow et al., 2015), processing and memory load (Chen, 1986; Gonnerman, 2012; Hawkins, 2004; Lohse et al., 2004; Szmrecsanyi et al., 2016), semantic and pragmatic considerations (Antomo & Steinbach, 2010; Krifka, 2004; Levin, 2015), and sociolinguistic considerations (Farrar, 1999), and also including information-theoretic considerations. A recent example of an information-theoretic approach is the notion of lossy-context surprisal (Futrell et al., 2020), which aims to combine information-theoretic approaches with processing-based approaches. However, we do not see enough evidence for hypothesizing that the desire to smooth the speech signal has a considerable effect on a speaker’s syntactic choices in a very general, pervasive sense. Signal smoothing does happen on other linguistic levels and arguably, the notion of information density is a useful concept for language perception.

The considerations above are fully compatible with the idea that communication is efficient and that syntax is optimized (Gibson et al., 2019; Hawkins, 1994, 2004; Zipf, 1949). The language facility could well be optimized toward factors such as a reduction of processing/memory load, as measured in dependency lengths, see Mollica et al. (2020), or as modeled with lossy-context surprisal (Futrell et al., 2020).

## ACKNOWLEDGMENTS

Many thanks to the reviewers for the excellent review process. Thanks also to Jana Häussler for the very thorough and challenging feedback, to Yuri Bizzoni for both the feedback and encouragement, and to Jörg Knappen for further feedback. Two other colleagues also provided extremely valuable feedback but wish to remain anonymous.

## DATA AVAILABILITY

Our data is available on GitHub (where possible qua license), alongside our script and additional plots: https://www.github.com/tjuzek/om-uid.

## Notes

^1^ Throughout this paper, “information” refers to the information theoretic notion, specifically surprisal, measured in bits, as per Shannon’s seminal work (Shannon, 1948). For linguistic data, surprisal is often based on syllables, lemmas, or actual word forms.^2^ To us, it is unclear if testing the hypothesis with a literal interpretation of the *ceteris paribus* is possible at all.^3^ For further issues with Δ-*UID* see Wulff et al. (2018, p. 108).^4^ Some of the constructions even allowed for more than two alternatives, but to keep our test setup simple, we only included pairs. Not including further alternatives is in the favor of the hypotheses, as including more variants increased the chance of a false negative.^5^ Hypothetical items marked as slightly less natural were still included, as this works in the models’ favor. Clearly, the models should prefer a real occurrence over a constructed alternative that is somewhat less natural.^6^ In fact, to make sure that the test pipeline works, we included a sanity check, which consisted of ungrammatical sentences and hypothetical grammatical counterparts, and grammatical sentences and hypothetical ungrammatical counterparts. For this test class, all hypotheses made good predictions. The Low-IC model made the best predictions with a perfect F_1_-score of 1.0. We did not include these data, because these ungrammatical-grammatical pairs are not real occurrences and not real choices speakers face.
